# Iatrogenic Botulism Following Unregulated Cosmetic Botulinum Toxin Injection

**DOI:** 10.7759/cureus.93517

**Published:** 2025-09-29

**Authors:** Abdul Basit

**Affiliations:** 1 Accident and Emergency, Hinchingbrooke Hospital, Huntingdon, GBR

**Keywords:** antitoxin therapy, cosmetic botulinum toxin injection, dysphagia, iatrogenic botulism, neuromuscular weakness, unlicensed cosmetic procedures

## Abstract

We report the case of a 35-year-old woman who developed progressive neuromuscular weakness, bulbar symptoms, and dysphagia following administration of unlicensed cosmetic botulinum toxin at home by a non-medical acquaintance. Her husband, who received injections from the same vial, was admitted to intensive care and required intubation. Our patient required close airway monitoring, antitoxin therapy, immunoglobulins, and multidisciplinary support, but did not require ventilatory support. She gradually improved and was discharged with ongoing outpatient neurology and speech therapy follow-up. This case highlights the dangers of unregulated access to botulinum toxin, the importance of early recognition of iatrogenic botulism, and the role of public health reporting in preventing further harm.

## Introduction

Early descriptions of botulism-like syndromes following botulinum toxin injections emphasized that systemic effects could occur after intramuscular administration [1]. Botulinum toxin treatment has spread incredibly fast over the past two decades worldwide, and it has become a very distinctive, minimally invasive aesthetic procedure that is done more than any other. It is simply a safe and effective toxin if it is administered by trained professionals in a medical setting using their approved formulation. As the demand for beauty enhancement has increased, so has the supply of illegal, counterfeit, or substandard products and services performed by street vendors [2,3]. Such practices have been linked to severe complications, including iatrogenic botulism, which, while rare, must be considered as a serious threat to public health because of its seriousness [4,5].

Iatrogenic botulism results when botulinum neurotoxin is inadvertently disseminated systemically after therapeutic or cosmetic injections. Older case reports have described botulism-like syndromes occurring after intramuscular injections for spasticity and aesthetic purposes - in these reports, a therapeutic product was used, but an unfavorable reaction occurred [1,3,6]. More recently, large outbreaks linked to counterfeit or illegally distributed formulations have been recorded, in high-income as well as middle-income countries, indicating the worldwide nature of the problem [7-9]. A travel-related outbreak in 2023, which spanned four European countries, was related to intragastric botulinum toxin injections for weight loss [8]. These incidents, along with others in Austria, Germany, Türkiye, and the United States, highlight the continuing threats posed by the presence of counterfeit or unlicensed products in the market [7,9,10].

Epidemiologic analyses and case series acknowledge that adverse events occur more commonly after injection procedures performed by non-medical practitioners or when products are purchased through unregulated online markets [11,12]. A recent retrospective study from China, along with case series released from Europe and the Middle East, identified multiple clusters of cosmetic iatrogenic botulism due to unlicensed injections, with presentations spanning cranial neuropathies to fulminant respiratory failure requiring mechanical ventilation [2,5,8,12]. These findings emphasize that counterfeit formulations and unsafe administration practices remain a persistent driver of iatrogenic cases worldwide.

Clinically, the disorder usually presents with progressive cranial nerve involvement, bulbar weakness, dysphagia, and autonomic dysfunction that, if not recognized early enough, rapidly compromises respiration [13,14]. Early recognition is key to determining the end outcome, followed by botulinum antitoxin treatment and supportive multidisciplinary care [15]. Individual reports have highlighted the importance of vigilance in cosmetic practice; however, recent literature increasingly shows that unregulated botulinum toxin use continues to cause needless morbidity and mortality.

This paper further adds to the available literature by providing evidence of a case of iatrogenic botulism following a cosmetic injection with a product purchased online, further amplifying the dangers unregulated access poses, the role of public health reporting, and the imperative of clinical vigilance. Given the potential for rapid deterioration, diagnostic vigilance and timely antitoxin administration are recommended in current guidance [15].

## Case presentation

**Figure 1:**
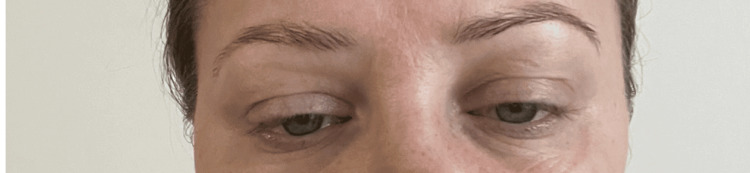
Bilateral ptosis on presentation Written informed consent to include this image in an online article was obtained from the patient.

A 35-year-old woman presented with five days of progressive facial weakness, hoarseness, dysphagia, and exertional shortness of breath. She had received cosmetic botulinum toxin injections to the forehead and periocular region seven days earlier, administered by a friend at home using a vial purchased online. Her husband, injected from the same vial, developed more rapid deterioration and required intubation and intensive therapy unit (ITU) admission. On examination, she was alert and oriented, with bilateral ptosis (Figure 1), dysphonia, xerostomia, reduced facial sensation, slow tongue movements, and a quiet hoarse voice. Cranial nerve assessment showed reduced sensation in the left trigeminal distribution and uvular deviation [6]. Limb strength was preserved, though she described fatigability. Peak expiratory flow was reduced (340 L/min; expected 430).

Routine blood tests were not much out of the ordinary. Inflammatory markers were normal, and so was the full blood count, with the exception of mild lymphopenia (1.3 × 10⁹/L). Everything else was normal, such as serum electrolytes, renal function test, liver function test, and coagulation screen. Neuroimmunological studies revealed that acetylcholine receptor antibodies were negative (<0.23 nmol/L), and ganglioside antibodies (GM1 and GQ1b) were also negative, thereby making the diagnosis of myasthenia gravis and Miller-Fisher variants of Guillain-Barré syndrome less likely. Magnesium and calcium levels were normal (Table 1).

**Table 1:** Summary of key laboratory and serological findings

Test	Result	Reference range	Interpretation
Acetylcholine receptor antibody	<0.23 nmol/L	0.00–0.50	Negative
GM1 antibody	Negative	–	Negative
GQ1b antibody	Negative	–	Negative
CRP	4 mg/L	<5	Normal
White cell count	6.7 ×10⁹/L	4.0–11.0	Normal
Lymphocytes	1.3 ×10⁹/L	1.4–4.8	Mildly low
Haemoglobin	126 g/L	115–165	Normal
Platelets	205 ×10⁹/L	150–400	Normal
Sodium	135 mmol/L	133–146	Normal
Potassium	3.9 mmol/L	3.5–5.3	Normal
Magnesium	0.81 mmol/L	0.70–1.00	Normal
Coagulation screen (PT/INR/APTT)	Within range	–	Normal

Serum samples taken before antitoxin administration were sent to the UK Health Security Agency, Colindale, for confirmation testing, and the implicated vial was sent to the MHRA. The product was later found to be an unlicensed imported preparation.

**Figure 2:**
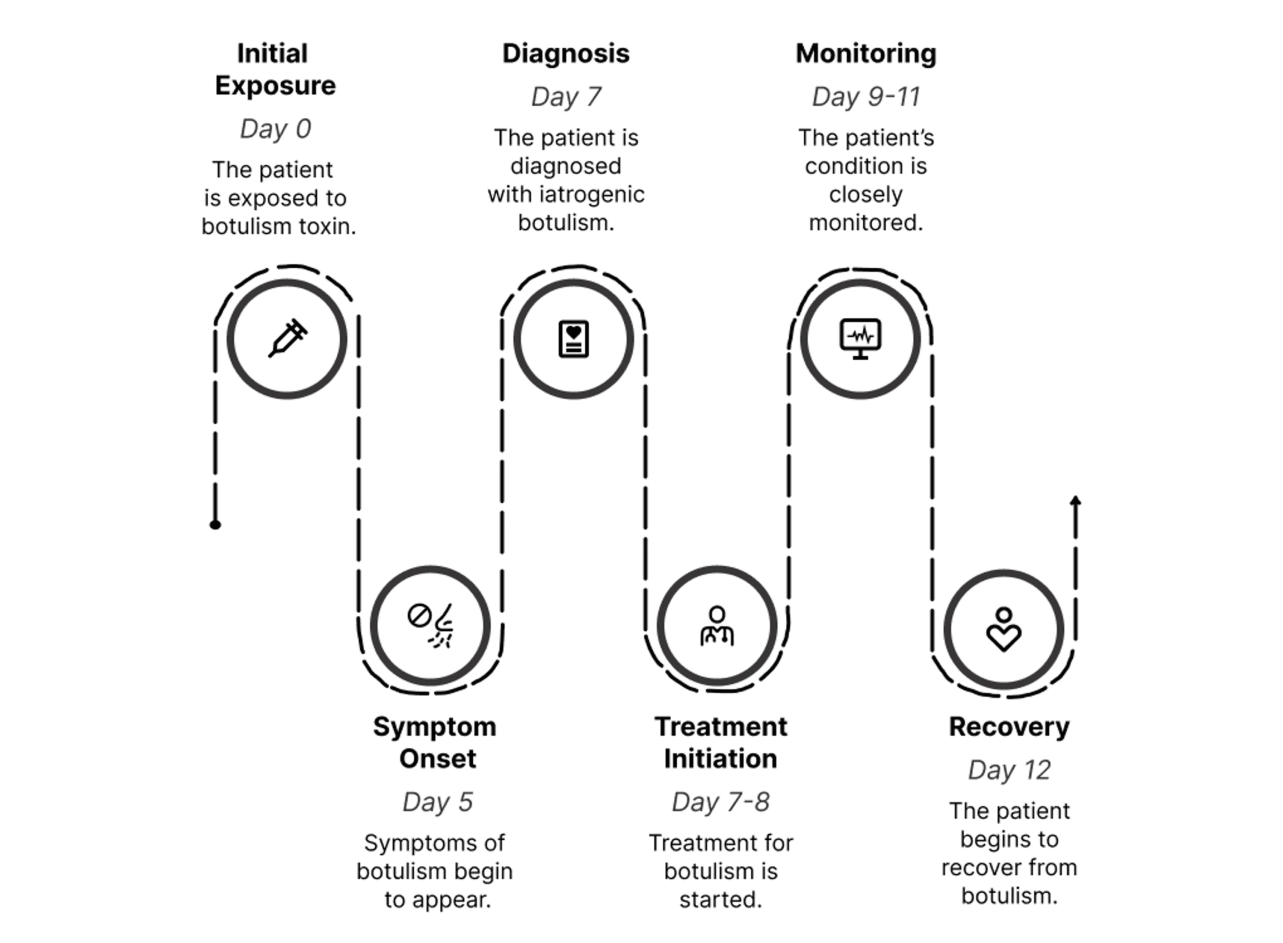
Clinical timeline of iatrogenic botulism case

The hospital course is summarised in Figure 2. She was monitored in the ITU and treated with botulinum antitoxin and intravenous immunoglobulin. She received symptomatic therapy, including artificial saliva, antihypertensives, and analgesia. She was reviewed by speech and language therapy, placed on a modified diet, and received physiotherapy and neurology input. Public health notification and case registration with the UK Health Security Agency were completed.

Swallowing function gradually improved, and she was able to tolerate soft food prior to discharge. She did not require mechanical ventilation. At discharge, she had mild residual ptosis, diplopia, and exertional fatigue. Outpatient follow-up was arranged with neurology and speech and language therapy. Her husband, who had initially required mechanical ventilation, also improved following treatment with antitoxin and intravenous immunoglobulin.

## Discussion

This case brings into view clinical and public health implications related to iatrogenic botulism due to cosmetic botulinum toxin injection in unlicensed circumstances. The patient had bilateral ptosis, dysphagia, xerostomia, and cranial neuropathies, all of which occurred relatively early in neurotoxin-mediated pathology and can swiftly proceed to bulbar and respiratory compromise in the absence of treatment [1,2,5]. While her husband had to be intubated, our patient benefited from early diagnosis, ICU-level monitoring, and timely antitoxin administration, which have been demonstrated to reduce morbidity and avert mortality [4,7,13].

Differential diagnoses included myasthenia gravis and the Miller-Fisher variants of Guillain-Barré syndrome. However, the negative acetylcholine receptor antibodies (<0.23 nmol/L) and the absent ganglioside antibodies (GM1, GQ1b) argued against these diagnoses. In addition, the normal inflammatory markers (CRP, 4 mg/L), an unremarkable full blood count apart from mild lymphopenia, and stable electrolytes pointed towards toxin-mediated rather than autoimmune or infective etiologies. Neurological examination noted during inpatient referral further supported a clinical picture of bilateral ptosis, patchy hyporeflexia, with preserved strength in limbs and no ophthalmoplegia - more suggestive of botulism instead of neuromuscular junction disorders (see Investigations). With these findings, the need for a structured diagnostic approach in situations where symptom overlap with other neuromuscular conditions could delay appropriate therapy was emphasized.

Put another way, recent studies stress the ill effects associated with counterfeit or unregulated botulinum toxin products. Sporadic outbreaks had emerged at different points in time in Europe and the Middle East in connection with intragastric injections and counterfeit brands purchased online [12,15,16]. Following intragastric injection for weight loss in 2023, a large travel-associated outbreak in Türkiye disseminated into many European countries [15]. Similarly, case series from Austria, Germany, and China documented clusters of cosmetic iatrogenic botulism requiring intensive care, showing dangerous patterns still active in various regions [16]. Cases in the U.S. also purportedly undermine health following the administration of counterfeit botulinum toxins in nonmedical facilities [12,17]. Taken together, these underscore the data indicating that counterfeit formulations remain on wide shelves for common use and are still a challenge to public international health.

What became of the patient illustrates the value of early involvement from multidisciplinary teams. There was close monitoring by the neurology and intensive care teams, while speech and language therapy was responsible for safe swallowing. The treatment given was artificial saliva for xerostomia and physiotherapy for residual fatigue. Importantly, the implicated vial was passed to the Medicines and Healthcare products Regulatory Agency (MHRA), and public notification was made to the UK Health Security Agency (UKHSA), an action sustained by international recommendations [13]. The measures help in confirming the case but also in preventing further exposures through regulation.

This case exemplifies variability in presentation and disease severity in patients exposed to the same vial, one requiring mechanical ventilation and the other remaining stable [4]. Variability in outcomes has also been reported in case series with host factors, dose, and injection technique involuntarily influencing clinical outcomes [9,10,18]. This patient recovered without ventilatory support, attesting that early diagnosis, rapid antitoxin administration, and careful monitoring can dramatically shift prognosis, even in severe iatrogenic cases. Public health messaging and awareness initiatives remain important adjuncts to clinical management to reduce future events [18].

## Conclusions

Clinicians should maintain a high index of suspicion for iatrogenic botulism in patients presenting with cranial neuropathies, bulbar weakness, or unexplained dysphagia, particularly if they have recently undergone cosmetic procedures. The early recognition of botulism, along with prompt diagnosis and intervention, can significantly improve prognosis and reduce the need for prolonged ventilatory support.

Public health reporting and the regulation of cosmetic procedures are essential to reducing the risks associated with unlicensed botulinum toxin products. This case serves as a reminder of the dangers of unregulated cosmetic practices and the importance of clinical vigilance in managing such potentially fatal complications.
